# CAPES publications portal: a decisive milepost in Brazilian scientific development providing sources of evidence for researchers, students and health professionals

**DOI:** 10.1590/S1516-31802007000400001

**Published:** 2007-07-01

Knowledge is one of the principal raw materials for development in all fields: for academic and professional training, research activities and healthcare decision-making. Sharing of information enables new knowledge to be generated, which causes growth-generating effects in quality research and technological development, with the effect of causing changes in the social context that is presented. The set of information available and found through portals may give rise to advances in all social spheres. Here, we highlight the CAPES publications portal (http://www.periodicos.capes.gov.br), which for users in Brazilian universities and participating institutions, and for general users, represents a milestone in educational development, research and technology in this country.

The CAPES publications portal originated in November 2000 as an initiative by a national consortium for access to electronic periodicals, under the auspices of the Ministry of Science and Technology.^1^ This portal is maintained by CAPES, which is a foundation within the Ministry of Education that invests in the development of *stricto sensu* postgraduate programs in our country. With the advent of this portal, Brazilian postgraduate and research programs have gained in quality, productivity and competitiveness.

The aim of the portal is to provide unrestricted access and use, free of charge to the participating institutions. It also provides some of this freely accessible content to all users via the internet.^1^

Access to this portal is made available to the teachers, researchers, students and employees of 188 higher education and research institutions throughout this country. This participation is distributed as follows:^2^

- 53 federal higher education institutions;
- 29 federal research institutes and units;
- 25 state and municipal public higher education institutions with postgraduate programs appraised by CAPES;
- 16 private higher education institutions with at least one staff member with a doctorate, with a three-yearly rating of five or over from CAPES;
- 33 federal technological education centers
- 32 institutions that have subscribed to the portal in the "paying" category, with access restricted to the publications contracted.

The sources of academic information that are freely accessible and available through the portal can be accessed by any internet user, whether or not linked to any participating institution.

In the year 2006, the CAPES publications portal experienced significant demand: 15 million complete-text articles downloaded and 32 million reference database consultations. The half-yearly statistics for the portal in 2007 show that 7.4 million articles have been downloaded and there have been 4.7 million reference database consultations.

## WHAT THE PORTAL OFFERS

The portal offers access to up-to-date scientific production from around the world, in all fields of knowledge. This is available in the form of:^3^

- 11,419 periodical publications from Brazil and around the world;
- 90 databases with abstracts of documents;
- Sources of academic information with free access on the internet for all types of users.

## PORTAL CONTENT FOR THE FIELD OF HEALTHCARE

The portal includes 13 publishers with **complete-text periodicals**, thus totaling 7,500 scientific journals in the field of healthcare alone. The publishers present within the portal are the following:^4,5^

- American Chemical Society (ACS)
- American Psychological Association (APA)
- Blackwell
- Cambridge University Press
- Ebsco
- Highwire Press
- Nature
- Ovid
- Oxford University Press
- Electronic Periodicals on Psychology (PePSIC)
- Science Direct Online
- Springer Verlag
- Wilson

Furthermore, the reference databases with published abstracts for the field of healthcare make available the best databases, covering all the worldwide literature, thereby making it possible to efficiently map out the evidence.

- Web of Science
- Biological Abstracts
- CINAHL (Cumulative Index to Nursing and Allied Health Literature) with Full Text
- Index of Latin American Science Journals: periodical
- Lilacs (Latin American and Caribbean Literature on Health Sciences)
- Medline (Medical Literature Analysis and Retrieval System Online)/PubMed (National Library of Medicine, United States). This can be accessed through the portal via the National Library of Medicine, Ovid or Bireme (Regional Library of Medicine)
- PsycINFO (American Psychological Association)
- CAplus (Chemical Abstracts Plus)
- SportDiscus
- Cochrane Library

By way of reference works for the collections of periodicals, the portal makes available important specialized texts for consultation, such as:

- MICROMEDEX Healthcare Series
- DISEASEDEX General Medicine System

Among **other research sources on the internet**, and complementing the collection of periodicals with complete texts and the reference databases, the portal includes a selection of important sources of scientific and academic information that are available free of charge on the internet, among which the following can be highlighted:

- Psycoloquy (American Psychological Association)
- SciELO (Scientific Electronic Library Online: 439 periodicals that are freely accessible on the internet, published in Brazil, Argentina, Chile, Colombia, Costa Rica, Cuba, Spain, Mexico, Portugal, Peru, Uruguay and Venezuela.
- CAPES thesis database: abstracts of more than 280,000 theses and dissertations.

It is important to highlight the content of the portal that is available via the **free access** icon of the initial periodicals page. This is a resource open to everyone and not just the participating institutions, i.e. all internet users have access to the content.

Through proper exploration of the rich resources offered by the CAPES portal, we are sure that this country's postgraduate and scientific research programs in the field of healthcare, and also its undergraduate programs, will gain in quality, productivity and competitiveness. Moreover, technological development in healthcare takes place through access to information and knowledge that are strategically decisive for Brazil to be able to stand shoulder-to-shoulder with the best countries in the world. Brazilian scientific production has been growing much faster than the pace of the investments and this is due, among various factors, to the action of the CAPES appraisal system and the positive influence of the CAPES portal. We all need to make use of this extremely important free resource and give political support for its continuation, which sometimes is threatened by those who do not understand the value of scientific knowledge in improving and developing any society of a serious nature. It should be borne in mind that this portal receives around 130,000 visits per day and more than 30 million every year!

Speaking of updating, the Fifteenth World Colloquium of the Cochrane Collaboration, *Evidence-based Healthcare for All*, is taking place in São Paulo from October 23 to 27, 2007 (http://www.colloquiumbrasil.info). The social and scientific programs are excellent. There will be a show featuring Mônica Salmaso, a tropical dinner and a soccer "match of the legends" of the Brazilian national team versus participants. The course on Technological Advances in Evidence-based Healthcare alone is worth twice the enrollment fee. Brazilians pay half price.

Free access to the Cochrane Library in Brazil, with around one million visits per year, is also funded by CAPES.
